# Does digital economy affect the happiness of older adults?

**DOI:** 10.3389/fpsyg.2026.1692681

**Published:** 2026-03-13

**Authors:** Zhendong Wu, Luhan Wang, Fuqi Ma, Jiongcheng Lu, George W. Leeson, Rujing Kong

**Affiliations:** 1 School of Law, Humanities and Sociology, Wuhan University of Technology, Wuhan, China; 2 School of Management, Jinan University, Guangzhou, China; 3 Global Business School for Health, University College London, London, United Kingdom; 4 Oxford Institute of Population Aging, Oxford University, Oxford, United Kingdom; 5 School of Applied Economics, Renmin University of China, Beijing, China

**Keywords:** digital divide, digital economy, happiness, mental health, older adults

## Abstract

**Introduction:**

The happiness of older adults is considered as an important indicator that affects their life satisfaction and mental health situation. The association between digital economy development and happiness among older adults remains unclear. This study aimed to investigate the association and mechanism.

**Methods:**

A total of 8,665 respondents aged 60 and above were collected from the 2020 China Longitudinal Aging Social Survey (CLASS). This study linked micro-level wellbeing measures to a city-level digital economy index that combines internet development indicators with the Peking University Digital Financial Inclusion Index. Ordered response models are estimated with extensive individual and city controls. This research also implemented an instrumental-variable strategy, complemented by propensity score matching and robustness checks.

**Results:**

Digital economy was positively associated with older adults' happiness (OR = 3.022, *P* < 0.01). The association was not significant among offline older adults, but was strong among internet users (OR = 6.456, *P* < 0.01). Under the digital usage divide, the association was stronger for older adults with high device proficiency (OR = 3.995, *P* < 0.01) and for those using the internet at least once per week (OR = 6.373, *P* < 0.05) or daily (OR = 5.646, *P* < 0.01). Under the digital skills divide, stronger associations were observed among older adults whose primary internet purpose was social activities (OR = 6.973, *P* < 0.01), information acquisition (OR = 4.315, *P* < 0.01), leisure and entertainment (OR = 3.561, *P* < 0.01), and investment and consumption (OR = 13.210, *P* < 0.01). Mechanism tests further indicated amplification through digital government (OR = 2.425, *P* < 0.01), social capital (OR = 1.130, *P* < 0.1), and improved access to health services (OR = 1.728, *P* < 0.01).

**Conclusion:**

This study underscores the importance and role of digital economy for happiness promotion among older adults. The findings imply that digital infrastructure expansion alone is unlikely to deliver inclusive wellbeing improvements. Narrowing access gaps and strengthening digital capabilities are critical to ensure that aging populations share in digital dividends. Policy should shift from facility building to digital skills and usage support.

## 1 Introduction

The rapid expansion of the digital economy and the process of population aging have emerged as two defining forces shaping contemporary social development. This dual transformation is particularly pronounced in China, which creates a distinctive and valuable setting for research. China has the world's largest older population and is aging at a fast pace. At the same time, its digital economy has become a leading driver of economic growth and social transformation, with both scale and dynamism among the highest globally. According to the latest statistics, China's population aged 60 and above exceeded 320 million in 2025; it accounts for more than 23% of the total population^1^ Over the same period, the digital economy has continued to expand rapidly, ranking second worldwide in size and leading in growth. The simultaneous acceleration and close interaction of aging and digitalization make China an essential and representative case for understanding how the digital era affects older adults' wellbeing.

In this context, examining the mechanisms and conditions through which the digital economy shapes older adults' happiness is essential for both national strategy and global knowledge. For China, the question directly relates to how the country responds proactively to population aging; as for the international community, it offers a meaningful reference for societies confronting the combined challenges of digital transformation and demographic aging. China could provide a rich empirical setting. Digital infrastructure has achieved wide coverage. Besides, digital government services have expanded rapidly to grassroots levels, and the broader digital ecosystem has become increasingly comprehensive. At the same time, structural inequalities in digital resources persist across access, usage, and capability. These inequalities are especially salient among older adults and create a locally distinctive digital divide. This study therefore uses Chinese data and context to identify the pathways through which digital development affects happiness. It also examines how the digital divide shapes and constrains these effects. The evidence deepen theoretical understanding of how digital dividends are distributed, while also informing inclusive digital development and targeted aging policies across countries.

Building on the 2020 China Longitudinal Aging Social Survey (CLASS), this study investigates how the digital economy influences older adults' happiness and explore the moderating role of the digital divide. The results show that digital economy development significantly increases older adults' happiness. The relationship operates mainly through three mediating channels, including digital government development, social capital accumulation, and improved health services. The moderating effects differ across dimensions of the digital divide. This study contributes in several ways. Prior work emphasizes micro-level factors such as individual internet use. In contrast, this study adopts a macro perspective by focusing on regional digital economy development and by examining how digital dividends translate into wellbeing outcomes. At the theoretical level, this study moves beyond a direct association. It incorporates the digital divide as a key moderator across access, usage, and skills. This identifies heterogeneous effects and clarify boundary conditions for older adults' digital benefits. Regarding the practical level, the three mediating channels tested in this study have clear policy relevance. They provide concrete directions for more precise, inclusive, and coordinated policies that link aging governance with digital transformation.

**Figure 1:**
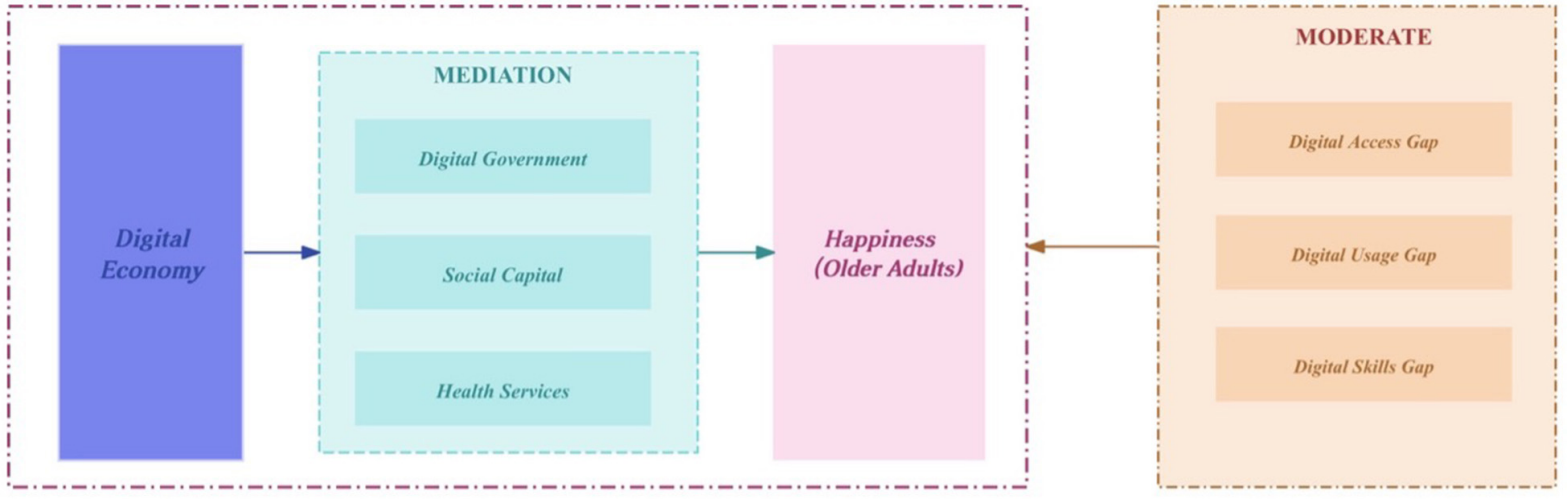
Research framework.

The remainder of this paper is organized as follows. Figure 1 presents the research framework. Section 2 reviews the literature and develops the theoretical framework. Section 3 describes the data, variable construction, and model specification. Section 4 reports the empirical results and presents mechanism and heterogeneity analyses. The final section summarizes the main findings and discusses policy implications.

## 2 Literature review and theoretical analysis

Most studies in happiness economics concentrated on the determinants. From a micro perspective, extensive discussions were made on the impact of socio-demographic characteristics, including gender, marital status, human-capital endowment, age, family assets, income, and social networks on individual happiness (Alesina et al., 2004; Cheung and Lucas, 2016; Di Tella et al., 2001; Oishi et al., 2011; Sanfey and Teksoz, 2007; Weaver and Habibov, 2012; Wolfers, 2003; Zagorski et al., 2014). Furthermore, scholars also explored the effects of macroeconomic factors, including regional economic growth, industry clusters, environmental pollution, and the quality of local healthcare (Easterlin, 1974, 1995; Grashof, 2025). However, few studies incorporated the digital economy into happiness economics.

The digital economy centers on digitized knowledge and information and is powered by core digital technologies. It speeds up technological upgrades and innovation, ties digital tools more closely to the real economy, and removes the barriers of distance and time in sharing and building knowledge. According to Han and Zhu (2014), the digital economy enabled the integration and processing of information, significantly reducing barriers to the flow of information, technology, and data across regions. Demonstration and spillover effects in the digital economy speed up technological progress across society, raising overall quality of life (Ren et al., 2024). Rapid advances in tools such as the internet have driven this expansion, and older adults feel its influence more than ever.

As for the theoretical perspective, the digital economy creates a more inclusive environment for older adults. It raises total factor productivity and improve regional economic structure. Wider digital infrastructure also lower barriers to participation. This helps reduce opportunity gaps relevant to age and geography (Zhao et al., 2023). Digital technologies act as a new factor of production. They reduce information costs and improve transaction efficiency (Xiao et al., 2025). This expands consumption choices and resource access for older adults. It then increases utility under a given budget constraint. Existing studies support these mechanisms. Regional digital economy development shapes individuals' psychological and behavioral outcomes. Higher digital economy development was associated with a lower likelihood of depression (Chen et al., 2024). Cross-country work found that higher national levels of digitalization predicted greater life satisfaction (Ionescu-Feleagă et al., 2022) and that frequent use of digital tools was linked to higher wellbeing (Kryzhanovskij et al., 2021). From existing evidence, urban digital economy development promotes internet access. It reshapes work and daily life, and raises subjective wellbeing (Sirgy et al., 1998). Under the “Internet +” agenda, the digital economy reshapes how everyday and social services are organized and how resources are allocated. This can deliver more inclusive and higher-quality products and services for groups such as older adults. The expansion of digital finance can improve their personal wellbeing (Kavetsos and Koutroumpis, 2011; Graham and Nikolova, 2013; Bruckner et al., 2017). Based on previous research, this paper proposes hypothesis 1: the digital economy's development significantly enhances older adults' happiness.

This study develops a multi-level and systematic theoretical framework to explain the mechanisms through which the digital economy influences older adults' happiness. It identifies digital government, social capital accumulation, and improvements in health services as the core mediating mechanisms. These three mechanisms form a coherent transmission chain across the institutional, social, and individual levels. At the macro level, digital government functions as an institutional public good. Its development responds directly to older adults' needs for safety, convenience, and fairness. This is consistent with Maslow's hierarchy of needs, particularly the basic demands for security and belonging. At the meso level, social capital accumulation is supported by the expanded social networks and communication modes enabled by the digital economy (Verwiebe and Hagemann, 2025). This reflects explanations from new economic geography and social identity theory; they emphasize how technology reshapes social connectedness. At the micro level, improvements in health services relate directly to older adults' physical conditions and access to medical care (Li and Li, 2025; Ding et al., 2024). This mechanism is grounded in health economics and technology acceptance perspectives. These mechanisms are complementary and mutually reinforcing in function. Digital government provides institutional support for the operation of social capital and the diffusion of health services. Social capital facilitates the spread of health information and the adoption of digital services. Improved health services enhance individual functioning, which supports social participation and strengthens institutional trust and identification.

With regard to the macro environment, digital economy, as an emerging economic form, is under the guidance and planning of the government. Since 2014, the Chinese central government has addressed the development of the digital economy in its annual government work reports. In 2018, the “Internet Plus Government Services” concept was included for the first time in the government work report. Digital government is a key form of digital dividends. It directly shapes the accessibility and efficiency of public services. From the perspective of Maslow's hierarchy of needs, digital government is particularly conducive to meeting older adults' security needs. Information flow theory points that the digital economy breaks down established information barriers. It accelerates information diffusion and lowers information acquisition costs, which in turn facilitates more interaction (Lu et al., 2024). Earlier studies reported that greater openness reduced corruption and strengthened trust in public order (Andersen, 2009; Li and An, 2020).

Moreover, the spread of smart security infrastructure, for example, CCTV networks, facial recognition checkpoints and big-data early-warning systems, provides real-time monitoring, lowers crime rates and heightens residents' sense of safety (Madsen et al., 2022). Additionally, integrated data platforms that link police, fire and medical records enabled rapid, coordinated responses; one-tap emergency apps shortened response times and raise satisfaction with local policing, which fed into higher overall happiness (Eom and Lee, 2022). Together with the lower administrative costs and richer information flows highlighted by Andersen (2009) and Lu et al. (2024), these features of digital government helped older adults access public services more smoothly, felt more secure and ultimately enjoyed greater happiness. Therefore, hypothesis 2 is presented: the development of the digital economy enhances the happiness of older adults by improving the construction of digital government.

According to the theory of new economic geography at the meso level, the digital economy eliminated the influence of geographic location on social capital through the globalization of information and services (Brynjolfsson and McAfee, 2014). Therefore, this study explores the pathways through which the digital economy affects the happiness of older adults from the perspective of social capital. The development of the digital economy offers a significant potential to enhance residents' social capital by consolidating existing social networks and fostering new social connections. Due to various objective factors such as work, relocation, time constraints, and retirement, the interpersonal relationships of older adults may weaken, leading to a deterioration of their real-life social networks. The advancement of the digital economy can break down spatial barriers, enabling online interactions and communication with friends and acquaintances. Specifically, the digital economy reduced the attrition of interpersonal networks caused by time and distance, thus compensating for offline social networks and promoting the accumulation of social capital (Kraut et al., 1998). Secondly, the enhancement theory emphasized the positive impact of internet use (Zhou et al., 2024). Compared to social network relationships established solely in offline environments, the digital economy era enables residents to use digital technologies to build new social relationships online and expand their social networks. Online socializing has become a popular choice for many older adults. The diverse online channels and information made it easier for them to find like-minded friends (Blanchflower, 2004). Previous researchers found that digital technology can impact residents' social interactions and the accumulation of social capital through networks, playing a positive role in happiness. Hence, this paper posits hypothesis 3: The advancement of the digital economy enhances the happiness of older adults by augmenting their social capital.

At the micro level, as the physical functions of older adults decline, obtaining sufficient health information and accessing timely medical services became essential for enhancing their sense of security (Pálsdóttir, 2011). Given the non-exclusivity and high penetration of the digital economy, it could notably impact health services. Therefore, this study explores the impact of the digital economy on the happiness of older adults through the mechanism of health services. When individuals perceive that digital technologies help them access information, engage in social interactions, or purchase goods more conveniently, they are more likely to adopt these technologies. Frequent participation in digital economic activities makes their life more convenient and enriching, which enhances their happiness. With the rapid growth of the digital economy, old adults turned to internet platforms as a means to broaden their access to information, enhancing cognitive functions and improving their capacity to acquire information and services (Luo et al., 2025). The diverse functionalities of the internet comprehensively cater to the varying levels and preferences of older adults, significantly boosting their satisfaction with life. One of the primary interests for older adults is health services. The internet enabled them to acquire health knowledge and proactively prevent or treat illnesses (Zagorski et al., 2014). Moreover, the rise of internet-based hospitals has alleviated older adults' difficulties obtaining medical appointments, particularly during pandemics. During this period, telemedicine provided older adults with medical and healthcare services, enabling them to access timely medical care (Feng et al., 2024). Online medical consultations offer a vital avenue for treatment, especially for those with mobility issues or limited access to transportation. Therefore, this study generates hypothesis 4: the advancement of the digital economy enhances the happiness level of older adults by improving the quality of health services.

Drawing on digital divide theory and technology adoption models, this study incorporates the digital divide into our analytical framework as a key moderator. This allows us to identify boundary conditions and subgroup heterogeneity in the relationship between the digital economy and older adults' happiness. Digital divide theory argues that systematic gaps exist across groups in access to information technologies, patterns of use, and the ability to translate technology into benefits. These gaps not only shape individuals' capacity to gain from digital development, but also alter the pathways through which the digital economy enhances social welfare. If the moderating role of the digital divide is ignored, studies will overstate the inclusiveness of digital dividends and obscure structural inequalities in their distribution. The older-adult digital divide refers to substantial differences in access to, adoption of, and skills for using information technologies among older people. These differences are shaped by socioeconomic status, education, and place of residence (Fidan, 2016). In this study, we conceptualize the digital divide along three dimensions, including access, usage, and skills. These dimensions shape how digital economy development affects happiness. They also condition the strength of the three pathways in our framework, like the institutional environment, social networks, and health services.

The digital access divide determines whether older adults can enter the digital environment. Without internet access, older adults are less able to use digital government services, engage in online social networks, or reach remote health resources. The channels through which the digital economy improves happiness are therefore weakened, or even blocked. The digital usage divide captures differences among those who are online. Older adults with higher device proficiency and more frequent use can operate digital tools more easily. They also engage with services more regularly. This makes it easier to convert local digital development into social support, health management capacity, and institutional trust; it further strengthens happiness gains. The digital skills divide concerns the ability to use the internet to achieve everyday goals. Older adults who use digital tools for socializing, learning, consumption, and entertainment expand the scope of social capital. They report higher self-efficacy and stronger social participation. These factors amplify the positive association between digital economy development and happiness.

We therefore propose the following hypothesis. As for Hypothesis 5a, the digital access divide weakens the positive effect of the digital economy on older adults' happiness. Compared with older adults who are offline, those who are online can access institutional benefits, social support, and health services enabled by digital development. The happiness gains from digital economy development are therefore stronger among older adults who are connected to the internet. Regarding hypothesis 5b, the digital usage divide strengthens the effect of the digital economy on older adults' happiness. Higher device proficiency and more frequent use improve the efficiency of benefiting from digital services. They support social capital accumulation and better health-service access. The positive association is stronger among older adults with deeper and more regular digital engagement. For Hypothesis 5c, the digital skills divide strengthens the effect of the digital economy on older adults' happiness. Older adults who use the internet for socializing, entertainment, information acquisition, and consumption are better able to translate digital technologies into practical resources and psychological rewards. The happiness gains associated with digital economy development are therefore stronger for those with broader and more purposeful digital use.

## 3 Materials and methods

Digital Economy indicators were obtained from two sources: (i) the China City Statistical Yearbook and (ii) the Digital Finance Inclusion Index compiled by the Peking University Institute of Digital Finance and Ant Financial Services Group. Micro-level information on older adults was taken from the 2020 wave of the China Longitudinal Aging Social Survey (CLASS). CLASS is a nationwide panel survey organized by the Institute of Gerontology at Renmin University of China and carried out by the China Survey and Data Center. It uses stratified multistage probability sampling, first selecting county-level units and then villages or neighborhood committees, and interviews residents aged 60 and above. The 2020 round was chosen since it is the most recent release and coincided with the COVID-19 pandemic, a period when digital technologies became central to daily life, work, and economic recovery. This design and data selection matches the study's focus. After excluding observations with missing values, refusals, or clear errors on key variables, 8,655 valid cases were retained for analysis.

### 3.1 Descriptions of variables

#### 3.1.1 Dependent variables

**Figure 2:**
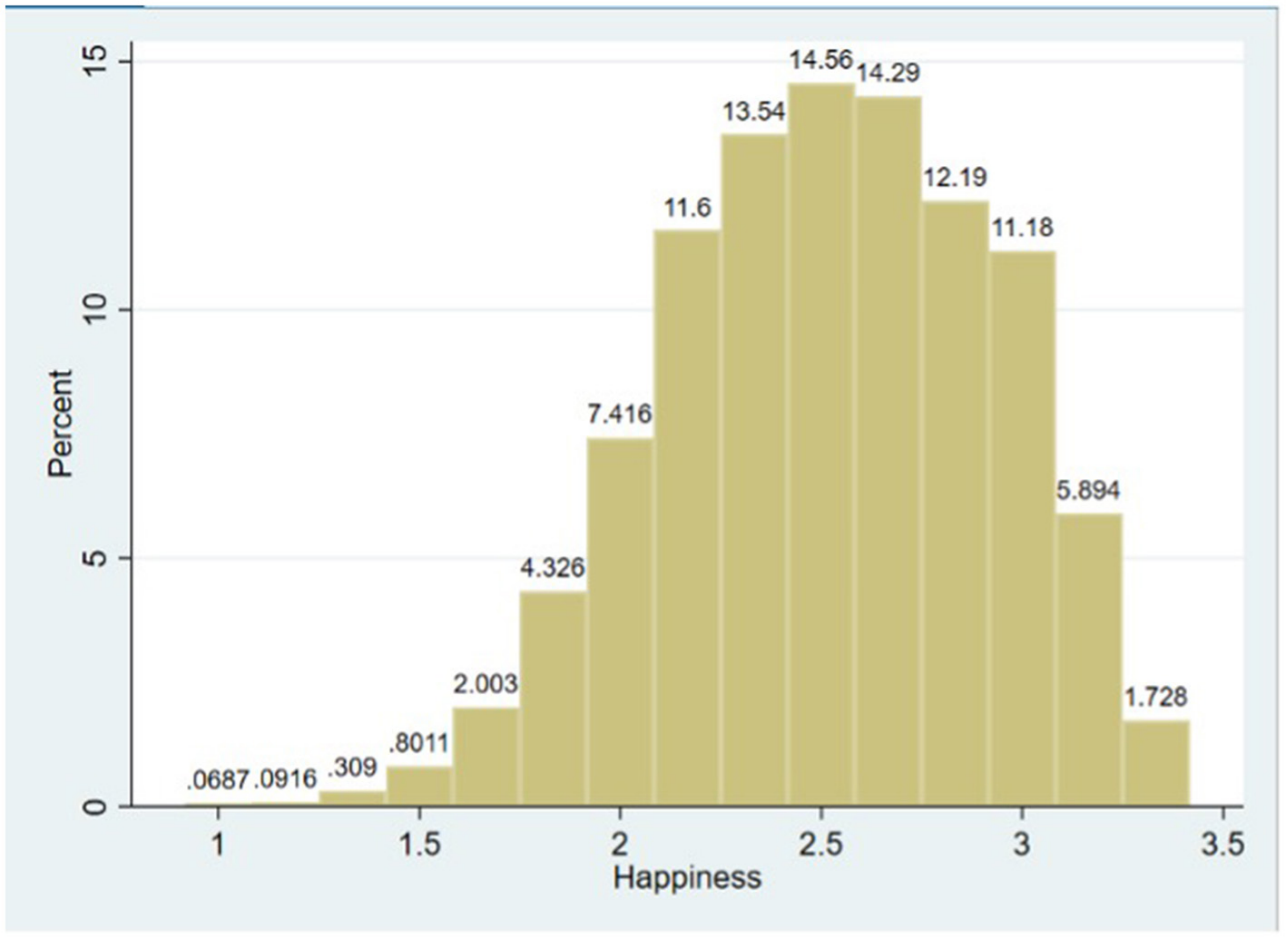
Distribution of happiness. Histogram with equal-width bins of 1/6; cases with missing, refusal, errors excluded.

Previous studies relied on single-variable measurements to gauge residents' happiness. In contrast, this paper, drawing inspiration from prior research (Andrews and McKennell, 1980), employs a multi-variable composite index to assess the happiness of older adults. Specifically, it utilizes responses to the following questions from a questionnaire to serve as measurement indicators: “Did you feel lonely in the past week?” “Did you feel very sad in the past week?” “Did you have poor sleep in the past week?” “Did you feel useless in the past week?” and “Overall, are you satisfied with your current life?” The answers to the first four questions are categorized as “often,” “sometimes,” or “never” and are assigned values of 1, 2, and 3, respectively. The fifth question is measured on a scale of “very dissatisfied,” “somewhat dissatisfied,” “neutral,” “somewhat satisfied,” and “very satisfied,” with corresponding values of 1, 2, 3, 4, and 5. Ultimately, the aggregate mean of these five variables is calculated to measure the happiness of older adults. This index integrates both positive and negative emotions to measure happiness. Compared to traditional “single-question” direct measurements, this method provides richer and more accurate content for assessment. Figure 2 illustrates the distribution of choices for the dependent variable *Happiness*.

#### 3.1.2 Independent variables

This study investigates how local digital economy development affects the happiness of older adults. In this paper, we explore the comprehensive development of the digital economy, denoted as *Digit*. The digital economy facilitated the digitization of transactions, communications, and collaborations through the internet, enhancing socioeconomic development (Andrews and McKennell, 1980; Barefoot et al., 2018; Pang and Zhu, 2013). The foundational internet access seems to be one of the determinants of local digital economy growth. Hence, this study considers the internet development level as one dimension of assessing the digital economy. In addition, the application of financial inclusion reflected the digital economy's development level and economic benefits (Ahmad et al., 2021; Zhao et al., 2020). This study adopts the digital finance inclusion index as another dimension for measuring the digital economy.

Furthermore, following the previous research (Tavares, 2020; Szeles and Simionescu, 2020), this study further constructs the digital economy indicators at the urban level and explores the impact of the digital economy on older adults' happiness; the urban-level digital economy directly or indirectly influences residents' work behavior and lifestyle, impacting individual happiness. The construction of variables at the urban level is primarily based on the role of urban infrastructure (5G networks, data centers, and industrial internet) in shaping digital economic growth, which drives the digital transformation of local services and influences individual experiences. This study focuses not on individual digitalization's impact on happiness but on whether regional digital economic development ensures shared benefits. It is particularly relevant for older adults, a vulnerable group, given global efforts to reduce digital inequality and address the challenges of population aging. Adopting the approach from Zhao et al. (2020), this study involves an assessment of the digital economy at the urban level in China.

Our assessment focuses on two key aspects: the development of the internet and the inclusiveness of digital finance. We measure internet development at the urban level, including four indicators: internet penetration rate, the proportion of employees in computer services and software industry relative to urban employed personnel, per capita telecommunications output, and mobile phone penetration rate per 100 people (Huang et al., 2019). The raw data for these indicators are sourced from the “China City Statistical Yearbook.” Regarding the development of digital finance, our study utilizes the Digital Financial Inclusion Index, a collaborative effort between the Institute of Digital Finance at Peking University and Ant Financial Services Group (Guo, 2020). Our study applies the principal component analysis to standardize and reduce the data dimensions from these five indicators, resulting in a comprehensive development index for the digital economy, which we call *Digit*. This research expands upon existing methodologies by measuring the extent of digital technology penetration and usage and considering the inclusiveness and accessibility of digital financial services. Our approach reflects the multidimensional nature of the digital economy and offers a more holistic view of its development at an urban level in China.

#### 3.1.3 Control variables

Based on the research focus of this paper, a range of socioeconomic characteristics were selected as control variables. These include variables such as religious affiliation (*Religion*), residential registration status (*Town*), political orientation (*Politic*), health condition (*Health*), marital status (*Marriage*), level of education (*Education*), living alone or not (*Family*), and gender (*Gender*). Since regional economic development could affect older adults' happiness as well, this study also controls for variables at the regional level, specifically using economic and social characteristics as control variables. Each of these variables potentially influences the outcomes studied and thus is vital for a comprehensive analysis.

These variables encompass diverse aspects of individuals' lives. For instance, religious affiliation (*Religion*) captures whether individuals identify with a religion, with the majority (94.79%) reporting no religious affiliation. Residential registration status (*Town*) reflects the urban-rural divide and is categorized as agricultural, non-agricultural, or transitioned to unified resident status, providing insights into individuals' socioeconomic backgrounds. Additionally, Political affiliation (*Politic*) distinguishes between Communist Party members and those affiliated with other or no political groups, highlighting potential ideological influences. Health condition (*Health*) is measured on a scale from very unhealthy to very healthy, capturing the physical wellbeing of respondents. Similarly, marital status (*Marriage*) accounts for key life circumstances, with most respondents either married or widowed. Level of Education (*Education*) spans levels from illiteracy to junior college, offering a nuanced understanding of individuals' intellectual resources. The variable “living alone or not” (*Family*) is a dummy variable and reflects whether respondents reside independently, a factor often associated with social support systems and mental health. Gender (*Gender*) is also included, with the dataset showing a near-even split between males and females, slightly favoring females (50.26%). Together, these variables provide a robust framework to account for potential confounding factors, as detailed in Table 1.

**Table 1:** Control variables.

**Variable names**	**Observations**	**Rate**
**Religious affiliation (** ***Religion*** **)**		
“Yes”	451	5.21%
“No”	8,204	94.79%
**Residential registration status (** ***Town*** **)**		
“Agricultural”	4,370	50.49%
“Non-agricultural”	3,881	44.84%
“Changed from agricultural to unified resident”	404	4.67%
**Political affiliation (** ***Politic*** **)**		
“Communist party member”	354	4.09%
“Democratic party member (including non-affiliated persons)”	8,299	95.91%
**Health condition (** ***Health*** **)**		
“Very unhealthy”	205	2.37%
“Relatively unhealthy”	1,058	12.22%
“Average”	3,134	36.21%
“Relatively healthy”	3,540	40.90%
“Very healthy”	718	8.30%
**Marital status (** ***Marriage*** **)**		
“Married with spouse”	6,626	76.56%
“Widowed”	2,029	23.44%
**Level of education (** ***Education*** **)**		
“Illiterate”	1,868	21.58%
“Private school/Literacy class”	357	4.12%
“Primary school”	3,143	36.31%
“Junior high school”	2,248	25.97%
“High school/Technical secondary school”	813	9.39%
“Junior college”	226	2.61%
**Living alone or not (** ***Family*** **)**		
“No”	7,861	90.83%
“Yes”	794	9.17%
**Gender (** ***Gender*** **)**		
“Male”	4,305	49.74%
“Female”	4,350	50.26%
Others	Sign	Measurement methods
Urban-level economic Development	GDP	Logarithm of GDP at the urban level
Urban-level cultural and entertainment environment	Entertainment	Logarithm of local government spending on culture and media
Urban-level regional healthcare environment	Medical_ins	Logarithm of the number of community health service centers

#### 3.1.4 Mediating variables

Based on the main results, hypotheses, and survey elements, the digital economy can directly influence the construction of digital government. For instance, the application of digital information technology significantly enhances public safety and increases the happiness of older adults. Moreover, the evolution of the digital economy has streamlined administrative tasks, particularly those handled by digitally proficient staff, thus improving service efficiency. This improvement brings convenience to older adults in their dealings with government services with more happiness. Consequently, this paper selects Digital Government as a mediating variable, with specific measurement indicators being individuals' satisfaction with the community's public safety environment and the committee's staff. Theoretically, the advancement of the digital economy should increase elderly citizens' satisfaction with community safety and staff, thus enhancing their happiness (Cuñado and De Gracia, 2013).

Furthermore, extensive research has been conducted on the impact of the digital economy on personal income. Many residents were either directly involved in the digital industry or benefited from the quality and efficiency improvements (Easterlin, 2001). Additionally, this study selects Social Capital as the second variable. The development of the digital economy could overcome geographical barriers, particularly through the rise of online platforms, bridging relationships neglected due to physical distance, and expanding social and friendship circles (Nordheim and Martinussen, 2020). The minimum number of friends or relatives an individual can meet or contact in a month is chosen to measure Social Capital. A higher number reflects stronger social connections and greater social capital. The digital economy consolidates and even enhances social capital, increasing the happiness of older adults. Lastly, Health Services are selected as another mediating variable. The emergence of internet hospitals provides convenience for online medical consultations for older adults. Health services were a crucial factor in assessing the happiness of older adults (Kotakorpi and Laamanen, 2010). This paper considers the impact of internet technology on health services for older adults as a measurement index. Theoretically, when older adults perceive a positive effect of internet technology on health services, they feel happier. Specific details can be found in Table 2.

**Table 2:** Mediators and moderators.

**Variable names**	**Observations**	**Rate**
**Digital government Satisfaction level with the public safety**		
**environment in the community**		
“Very dissatisfied” = 1	45	0.52%
“Somewhat dissatisfied” = 2	274	3.17%
“Neutral” = 3	2,274	26.27%
“Somewhat satisfied” = 4	3,997	46.18%
“Very satisfied” = 5	2,065	23.86%
**Satisfaction level with the capability of the committee staff**		
**in the community**		
“Very dissatisfied” = 1	32	0.37%
“Somewhat dissatisfied” = 2	336	3.88%
“Neutral” = 3	2,462	28.45%
“Somewhat satisfied” = 4	4,211	48.65%
“Very satisfied” = 5	1614	18.65%
**Social capital Family/Relatives (“How many family)**		
**members/relatives can you at least meet or contact”)**		
**in a month?”)**		
None	193	2.23%
One	952	11.00%
Two	2,374	27.43%
Three	3,561	41.14%
Four	1,234	14.26%
Five	341	3.94%
**Friends (“How many friends can you at least meet or contact)**		
**in a month?”)**		
None	443	5.12%
One	1,177	13.60%
Two	2,775	32.06%
Three	2,695	31.14%
Four	1,091	32.61%
Five	474	5.48%
**Health services “What impact has the commonly used**		
**internet technology in society brought to the health services**		
**in your life?”**		
“Inconvenient” = 1	1,757	20.30%
“No impact” = 2	2,708	31.29%
“Indeterminate” = 3	1,893	21.87%
“Convenient” = 4	2,297	26.54%
**Digital access gap (Internet usage)**		
“Not using the internet” = 0	6,158	71.15%
“Using the internet” = 1	2,497	28.85%
**Digital usage gap (Proficiency with electronic devices)**		
“Low proficiency” = 0	1,057	12.21%
“High proficiency” = 1	7,598	87.79%
**Digital usage gap (Internet usage frequency)**		
“A few times a year” = 0	48	1.92%
“At least once a month” = 1	71	2.84%
“At least once a week” = 2	491	19.66%
“Daily” = 3	1,887	75.57%
**Digital skills gap (Purposes of internet use)**		
“Social activities”	2,342	35.85%
“Information acquisition”	1,902	29.12%
“Entertainment and leisure”	1,544	23.64%
“Investment and consumption”	744	11.39%

#### 3.1.5 Moderate variables

This study treats the digital divide as a moderating variable and follows earlier research in distinguishing three dimensions. The digital access divide referred to whether an individual is connected to the internet (Dewan and Riggins, 2005). The digital usage divide captured differences that arise during actual use and is measured by proficiency with electronic devices and the frequency of internet use. The digital skills divide reflected variation in the ability to engage productively online (Scheerder et al., 2017). Moreover, if older adults remain offline, they cannot share in the digital dividend. Even when they go online, limited device proficiency and infrequent use could reduce the benefits they gain from local digital economy growth; insufficient digital literacy could further constrain their participation in digital production and commerce (Wang et al., 2022). To quantify these divides, the study employs four indicators: internet connection status, device proficiency, internet use frequency, and primary purpose of internet use. Coding details for each measure are reported in Table 2.

### 3.2 Model selection

To further analyse the digital economy's impact on older adults' happiness, this study mainly constructs the following Ordered-logit model (Li and Li, 2025; Zhang et al., 2024) (1). The model (1) represents both the Ordered Logit and Ordered Probit frameworks. The key difference between these two lies in their assumptions about the distribution of the error term: the Ordered Logit model assumes a logistic distribution. In contrast, the Ordered Probit model assumes a standard normal distribution. The latter is applied to ensure robustness in the analysis.


$$\begin{array}{l} {\text{Happiness}}_{i}=\alpha_{0}+\alpha_{1}{\text{Digit}}_{c}+\sum \text{Controls}+\varepsilon \end{array}$$ (1)

In this study, we examine the relationship between individual happiness and the level of digital economic development in their residing cities. Specifically, the variable “*Happiness*_*i*_” represents the happiness of individual *i*, while “*Digit*_*c*_” denotes the degree of development of the digital economy in city *c* where individual *i* resides. Additionally, this study controls for a range of both urban characteristics and personal variables, *Controls*. A central aspect of our analysis focuses on the coefficient α_1_, which assesses the impact of digital economy development on individual happiness. The sign and statistical significance of α_1_ are of particular interest. If this coefficient is consistently positive and significant, it suggests that the advancement of the digital economy positively correlates with the enhancement of older adults' happiness. These findings could imply that digital economic growth could be a crucial factor in improving the quality of life for the elderly population.

## 4 Results

### 4.1 The digital economy & older adults' happiness

As Table 3 demonstrates, there is a significant correlation between the digital economy and the happiness of older adults (1.106, *p* < 0.01). A one-unit increase in digital economy development raises the log-odds of older adults reporting a higher happiness category by about 1.106, which supports Hypothesis 1. The digital economy, driven by innovative technologies such as cloud computing, blockchain, and big data, improved the efficiency of factor allocation and optimized resource distribution (Bai and Zhang, 2021). It has brought positive changes to older adults' lifestyles and work patterns. New forms such as remote work, online shopping, online entertainment, and the gig economy have helped older adults integrate into the digital economy era. Additionally, the regression coefficient for the variable *Religion* is significantly negative, indicating that older adults with religious beliefs tend to have relatively higher happiness. The variable *Politic* is also considerably negative, suggesting that older adults with a political status as the “general public” exhibit more substantial happiness. Variables *Health, Education*, and *Medical_ins* are significantly positive, implying that better health, higher levels of education, and better urban medical and health environments are associated with greater happiness among older adults. Conversely, variables *Marriage, Family, GDP*, and *Entertainment* are significantly negative. The results indicate that older adults who are widowed, living alone, and residing in areas with high economic development and better entertainment environments tend to experience lower happiness. Household registration type and gender do not have a significant impact on the happiness of older adults.

**Table 3:** Ordered-logit model approach to digital economy's influence.

**Variables**	**(1)**
	**Happiness**
*Digit*	1.106^***^
	(6.268)
*Religion*	−0.234^**^
	(−2.539)
*Politic*	−0.121^**^
	(−2.561)
*Health*	0.736^***^
	(29.734)
*Town*	−0.057
	(−1.498)
*Education*	0.157^***^
	(9.418)
*Marriage*	−0.373^***^
	(−6.920)
*Family*	−0.177^**^
	(−2.268)
*Gender*	0.022
	(0.562)
*GDP*	−0.162^**^
	(−2.386)
*Entertainment*	−0.228^***^
	(−3.305)
*Medical_ins*	0.428^***^
	(7.753)
Observations	8,665
Pseudo R2	0.039

Robust t-statistics in parentheses.

^***^p < 0.01, ^**^p < 0.05, ^*^p < 0.1.

### 4.2 Robustness

Due to the use of cross-sectional data in this study, there exists a non-negligible endogeneity issue in the relationship between the digital economy and happiness. To ensure the robustness of our main regression results, we adopted several strategies informed. We employed an instrumental variable approach to mitigate potential reverse causality (Wooldridge, 2010; Angrist and Pischke, 2009). Furthermore, we conducted additional robustness checks by utilizing alternative regression models and incorporating additional control variables to address concerns related to omitted variable bias. Collectively, these methodological efforts enhance the plausibility of a causal interpretation of our findings (Pearl, 2009).

#### 4.2.1 Instrumental variables

Drawing on previous literature (Zou and Pan, 2023), this study selects the interaction between the number of post offices per million people in 1984 and the revenue of China's information technology services, as well as the interaction between the number of fixed-line telephones per million people in 1984 and the revenue of China's IT services, as instrumental variables (Dou et al., 2023). On one hand, the construction and improvement of urban telecommunication and network infrastructure are essential foundations for digital economic development. Chinese internet initially relied on dial-up connections. Cities with historically higher numbers of fixed-line telephones are likely to have higher levels of digital economic growth, meeting the relevance condition for instrumental variables. On the other hand, the number of post offices and the number of fixed-line telephones owned in 1984 are historical data. The impact of traditional communication and telecommunication tools on economic development has gradually diminished as their usage frequency has decreased, making it unlikely to affect the happiness of current older adults. This satisfies the exogeneity condition of the instrumental variable (Li et al., 2021). As shown in Table 4, the instrumental variable regression results in column (1) confirm the robustness of the main regression findings of this paper. Column (2) presents the regression results after Propensity Score Matching (PSM). Moreover, the significance of the control variables shows no noticeable difference compared to the baseline regression. Subsequently, this study employs the Ordered Probit model for further robustness tests.

**Table 4:** Instrumental variable adoption.

**Variables**	**(1)**	**(2)**
	***Happiness***	***Happiness***
*Digit*	1.023^***^	1.904^***^
	(16.591)	(6.886)
*Religion*	−0.071^***^	−0.396^**^
	(−3.593)	(−2.282)
*Politic*	−0.031^***^	−0.038
	(−3.022)	(−0.458)
*Health*	0.161^***^	0.703^***^
	(31.516)	(18.517)
*Town*	−0.046^***^	−0.087
	(−5.556)	(−1.620)
*Education*	0.019^***^	0.158^***^
	(5.319)	(6.587)
*Marriage*	−0.083^***^	−0.325^***^
	(−6.954)	(−4.250)
*Family*	−0.027	−0.344^***^
	(−1.574)	(−3.028)
*Gender*	0.005	0.086
	(0.564)	(1.455)
*GDP*	−0.073^***^	0.061
	(−4.717)	(0.571)
*Entertainment*	−0.137^***^	−0.456^***^
	(−8.541)	(−4.640)
*Medical_ins*	0.146^***^	0.535^***^
	(11.451)	(5.818)
Observations	8,665	4,195
Pseudo R2	0.124	0.035

Robust t-statistics in parentheses.

^***^p < 0.01, ^**^p < 0.05, ^*^p < 0.1.

#### 4.2.2 Alternative model & independent variables

Subsequently, this study employs the Ordered Probit model for further regression testing, as indicated in the first column of Table 5. Furthermore, this study applies the entropy weight method to standardize and reduce the dimensionality of the five indicators for a comprehensive digital economy development index^2^. The regression analysis, as shown in Table 5 column (2), confirms the robustness of the study's findings. Furthermore, the significance of the control variables in the three regressions in Table 5 is largely consistent with that of the baseline regression. Simultaneously, the study also incorporates a set of control variables outlined in the third column of Table 5. The variables under consideration include the health status of parents when the child reaches the age of 10 (*Parents*), the economic standing relative to others (*Econ*), the availability of childhood healthcare services (*Medical*), instances of food scarcity (*Hunger*), a comparative analysis of recent health issues compared with the previous year (*Health2*), chronic health conditions in relation to contemporaries of the same age (*Health1*), and the age of the spouse (*Age2*). The results remain robust, indicating that the digital economy can enhance the happiness of older adults.

**Table 5:** Robustness tests.

**Variables**	**(1)**	**(2)**	**(3)**
	***Happiness***	***Happiness***	***Happiness***
*Digit*	0.589^***^	0.703^***^	1.021^***^
	(5.929)	(6.178)	(5.804)
*Religion*	−0.146^***^	−0.104^**^	−0.266^***^
	(−2.868)	(−2.028)	(−2.902)
*Politic*	−0.063^**^	−0.065^**^	−0.123^***^
	(−2.259)	(−2.352)	(−2.677)
*Health*	0.430^***^	0.433^***^	0.531^***^
	(30.790)	(30.695)	(16.690)
*Town*	−0.032	−0.035^*^	−0.043
	(−1.521)	(−1.651)	(−1.140)
*Education*	0.092^***^	0.088^***^	0.110^***^
	(9.748)	(9.301)	(6.491)
*Marriage*	−0.210^***^	−0.210^***^	−0.267^***^
	(−6.757)	(−6.764)	(−4.756)
*Family*	−0.120^***^	−0.120^***^	−0.266^***^
	(−2.678)	(−2.679)	(−3.429)
*Gender*	0.020	0.019	0.047
	(0.883)	(0.842)	(1.163)
*GDP*	−0.028	−0.015	−0.168^**^
	(−0.711)	(−0.396)	(−2.412)
*Entertainment*	−0.177^***^	−0.228^***^	−0.178^**^
	(−4.442)	(−5.375)	(−2.533)
*Medical_ins*	0.208^***^	0.161^***^	0.572^***^
	(6.560)	(5.224)	(11.014)
*Parents*			0.184^***^
			(4.365)
*Medical*			0.421^***^
			(7.348)
*Hunger*			−0.132^***^
			(−3.105)
*Econ*			0.060^*^
			(1.665)
*Health2*			−0.052
			(−1.058)
*Health1*			0.303^***^
			(8.258)
*Age2*			−0.014^***^
			(−4.694)
Observations	8,665	8,655	8,665
R2	0.041	0.046	0.046

Robust t-statistics in parentheses.

^***^p < 0.01, ^**^p < 0.05, ^*^p < 0.1.

### 4.3 Mediation & moderation analysis

#### 4.3.1 Moderation analysis

The digital economy has revolutionized the way of life, notably through the advent of intelligent technologies. These innovations, ranging from autonomous vehicles and robots to the burgeoning virtual economy, significantly contribute to productivity and societal progress. However, the digital realm also presents its own set of challenges. The inherent digital divide, along with the polarizing effects of new technological applications, causes societal rifts (Peña-López, 2007). The digital divide, as one of the digital inequities, could significantly impact the happiness of older adults. The digital divide among older adults referred to the significant disparities in access to, adoption of, and proficiency with information technology within this group, driven by differences in socioeconomic status, educational attainment, and geographic location (Pénard et al., 2013).

Based on this study, the digital divide is categorized into three types: the “Digital Access Gap,” referring to whether individuals have internet access; the “Digital Usage Gap,” referring to differences in how the internet is used; and the “Digital Skills Gap,” referring to variations in the skills required to use the internet effectively. This paper delves into how the digital economy influences the happiness of older adults, particularly in the context of multiple dimensions of the digital divide. Table 6 provides a detailed view of how the digital economy affects older adults' happiness. Columns (1) and (2) report ordered-logit estimates of the digital economy variable under two groups. Among older adults who are offline, the coefficient is 0.085 and insignificant (*p* > 0.10), indicating no clear gain in happiness. Among those who use the internet, the coefficient rises to 1.865 and is strongly significant (*p* < 0.001), showing a sizeable increase in happiness. The digital economy, therefore, raises happiness only for online older adults. As Pénard et al. (2013) argued, the most direct channel through which digital development benefits seniors was encouraging them to connect to the internet. Those who remain offline miss timely information and lack opportunities to learn digital skills, which slows the diffusion of digital services in this age group. By contrast, connected seniors can participate in a wide range of online activities and so capture the happiness gains associated with local digital progress. The results make clear that the digital access divide limits older adults' ability to share in the dividends of the digital economy, which corresponds to hypothesis 5a.

**Table 6:** Moderating effect of the multidimensional digital divide.

**Variables**	**(1)**	**(2)**	**(3)**	**(4)**	**(5)**	**(6)**	**(7)**	**(8)**	**(9)**	**(10)**	**(11)**	**(12)**
	**Offline**	**Internet**	**High proficiency**	**Low proficiency**	**Multiple times per Year**	**At least once per month**	**At least once per week**	**Daily**	**Social Activities**	**Inform-ation acquisition**	**Leisure**	**Invest-ment**
	***Happiness***	***Happiness***	***Happiness***	***Happiness***	***Happiness***	***Happiness***	***Happiness***	***Happiness***	***Happiness***	***Happiness***	***Happiness***	***Happiness***
*Digit*	0.085	1.865^***^	1.385^***^	−0.377	3.583	−0.914	1.852^**^	1.731^***^	1.942^***^	1.462^***^	1.270^***^	2.581^***^
	(0.387)	(5.636)	(7.257)	(−0.757)	(0.865)	(−0.434)	(2.383)	(4.399)	(5.661)	(3.742)	(2.801)	(3.649)
*Religion*	−0.223^**^	−0.163	−0.143	−0.709^***^	−4.044^**^	−0.099	−1.490^***^	0.310	−0.081	0.301	−0.137	0.129
	(−2.002)	(−0.940)	(−1.417)	(−2.914)	(−2.188)	(−0.084)	(−4.080)	(1.576)	(−0.449)	(1.360)	(−0.650)	(0.479)
*Politic*	−0.072	−0.148^**^	−0.113^**^	−0.247^*^	−1.286^*^	−0.269	0.196	−0.215^***^	−0.166^**^	−0.113	−0.086	−0.227^*^
	(−1.076)	(−2.164)	(−2.242)	(−1.897)	(−1.729)	(−0.379)	(1.095)	(−2.860)	(−2.375)	(−1.442)	(−0.961)	(−1.886)
*Health*	0.651^***^	0.859^***^	0.731^***^	0.861^***^	1.127^**^	0.979^***^	0.828^***^	0.852^***^	0.890^***^	0.868^***^	0.853^***^	0.879^***^
	(22.225)	(18.055)	(27.306)	(12.038)	(2.393)	(3.482)	(8.947)	(14.472)	(17.871)	(14.644)	(13.980)	(8.547)
*Town*	−0.084^**^	−0.129	−0.003	−0.490^***^	0.758	0.383	−0.340^*^	−0.054	−0.119	−0.095	−0.217^**^	0.455^**^
	(−1.966)	(−1.570)	(−0.075)	(−4.067)	(1.192)	(0.619)	(−1.865)	(−0.544)	(−1.372)	(−0.939)	(−2.051)	(2.509)
*Education*	0.103^***^	0.145^***^	0.165^***^	−0.003	−0.542	0.182	0.088	0.162^***^	0.126^***^	0.179^***^	0.188^***^	0.187^***^
	(5.300)	(3.956)	(9.413)	(−0.042)	(−1.348)	(0.481)	(1.133)	(3.732)	(3.201)	(4.088)	(4.083)	(2.842)
*Marriage*	−0.314^***^	−0.324^**^	−0.351^***^	−0.735^***^	−2.499^**^	−0.205	−0.618^**^	−0.142	−0.304^**^	−0.316^**^	−0.409^**^	−0.258
	(−5.317)	(−2.363)	(−6.260)	(−3.500)	(−2.183)	(−0.341)	(−1.974)	(−0.884)	(−2.178)	(−2.022)	(−2.128)	(−0.995)
*Family*	−0.247^***^	−0.093	−0.224^***^	0.491^*^	0.158	−1.062	0.266	−0.179	−0.061	−0.019	−0.060	0.178
	(−2.898)	(−0.477)	(−2.766)	(1.661)	(0.142)	(−1.210)	(0.647)	(−0.768)	(−0.303)	(−0.086)	(−0.220)	(0.491)
*Gender*	0.042	−0.071	0.058	−0.248^**^	−1.324^*^	0.109	−0.042	−0.034	−0.069	−0.054	−0.042	0.086
	(0.885)	(−0.979)	(1.378)	(−2.208)	(−1.759)	(0.220)	(−0.248)	(−0.408)	(−0.918)	(−0.654)	(−0.456)	(0.655)
*GDP*	0.048	−0.422^***^	−0.080	−0.389^*^	−0.548	−0.607	−0.245	−0.338^**^	−0.397^***^	−0.857^***^	−0.446^***^	0.172
	(0.567)	(−3.234)	(−1.080)	(−1.939)	(−0.730)	(−0.585)	(−0.702)	(−2.226)	(−2.887)	(−5.450)	(−2.584)	(0.605)
*Entertainment*	−0.487^***^	−0.005	−0.348^***^	0.147	−0.348	0.576	−0.350	−0.017	−0.050	0.286^*^	0.103	−0.301
	(−5.508)	(−0.036)	(−4.539)	(0.729)	(−0.324)	(0.540)	(−1.076)	(−0.113)	(−0.359)	(1.900)	(0.641)	(−1.199)
*Medical_ins*	0.301^***^	0.654^***^	0.382^***^	0.474^***^	1.354	0.063	0.037	0.765^***^	0.677^***^	0.823^***^	0.719^***^	0.130
	(4.573)	(5.916)	(6.454)	(2.903)	(1.583)	(0.048)	(0.111)	(6.223)	(5.792)	(6.365)	(5.152)	(0.517)
Observations	6,158	2,497	7,598	1,057	48	71	491	1,887	2,584	1,902	1,544	744
Pseudo R2	0.030	0.045	0.040	0.048	0.153	0.078	0.054	0.043	0.046	0.043	0.046	0.044

Robust t-statistics in parentheses.

^***^p < 0.01, ^**^p < 0.05, ^*^p < 0.1.

After accounting for digital access, this study further examines how older adults use the digital economy. Hence, this study uses survey questions related to internet usage among older adults to explore the impact of the digital divide further. Columns (3) and (4) measure the proficiency of elderly internet users, while (5) through (8) assess their frequency of use. This study refers to them as the “Digital Usage Gap.” The findings reveal a clear correlation: older adults with higher proficiency in electronic devices are more likely to derive happiness from the digital economy, with a coefficient of 1.385 (*p* < 0.01). Older adults who are more proficient with electronic devices can experience the positive impacts of the digital economy on their daily lives, entertainment, and work. The frequency of internet use has a heterogeneous effect on the happiness of older adults. High-frequency usage, such as *Daily* (1.731, *p* < 0.01) or *at least* once *per Week* (1.852, *p* < 0.05), significantly enhances their sense of happiness. It indicates that older adults who are more proficient with electronic devices and use them more frequently are positioned to enjoy the benefits of the digital economy, improving their happiness. The digital usage divide can influence the extent to which the digital economy affects the happiness of older adults, proving the hypothesis 5b.

The analysis then turns to the contexts in which older adults apply their digital skills. Results from columns 9 to 12, highlight the “Digital Skills Gap.” Older adults who use the internet for social activities (coefficient 4.776, *p* < 0.01), leisure and entertainment (coefficient 5.214, *p* < 0.01), information acquisition (coefficient 4.895, *p* < 0.01), and investment (coefficient 2.581, *p* < 0.01) gain more happiness from the digital economy. These findings underscore the impacts of the digital economy on the happiness of older adults within multiple dimensions of the digital divide. The digital economy has enriched leisure activities such as self-media videos, online music, and internet games, with more fun added to their daily lives, enhancing older adults' happiness. Additionally, the digital economy helps overcome geographical barriers and reduce information asymmetry. It enables older adults to access information more quickly and conveniently than the relatively isolated information environment of the past, improving their happiness. Moreover, the digital economy introduced more financial investment tools, alleviating financing constraints and creating new opportunities for older adults to use financial products and invest; it also increased their savings and income and expanded consumption possibilities (Oishi et al., 2011), supporting the hypothesis 5c.

#### 4.3.2 Mediation analysis

After testing the moderate variables shown above, this section focuses on channels through which the digital economy improves happiness. Initially, this study proposed that the digital economy enhances older adults' happiness through digital government initiatives. Empirical results from Table 7 Column (1) indicate a positive influence (coefficient 0.886, *p* < 0.05) from the interaction part of the digital economy and digital government, which proves Hypothesis 2. It suggests that digital government, as a mechanism variable, supports the effect of the digital economy on the happiness of older adults. The digital economy has increasingly penetrated various industries, particularly government services, bringing greater convenience and efficiency to public welfare. Digital government initiatives can establish innovative elderly care platforms, break down data silos in civil affairs departments, and drive key reforms such as system restructuring, digital empowerment, and the digitalization of civil affairs services. These initiatives provided enhanced information services for older adults and elderly care institutions while offering better decision-making support for government management, ultimately improving the quality of elderly care services (Madsen et al., 2022). For instance, smart communities simplify personal tasks for older adults, reduce processing times, improve service efficiency, and enhance their happiness.

**Table 7:** Mediation test.

**Variables**	**(1)**	**(2)**	**(3)**
	***Digital government***	***Social capital***	***Health services***
	**Happiness**	**Happiness**	**Happiness**
*Digit*×*Digital Government*	0.886^***^		
	(3.971)		
*Digital Government*	0.122^*^		
	(1.819)		
*Digit*×*Social Capital*		0.122^*^	
		(2.691)	
*Social Capital*		0.247^***^	
		(5.476)	
*Digit*×*Health Services*			0.547^***^
			(4.046)
*Health Services*			−0.007
			(−0.178)
*Digit*	−2.728^***^	1.526^***^	−0.272
	(−2.991)	(3.145)	(−0.718)
*Religion*	−0.250^***^	−0.206^**^	−0.224^**^
	(−2.669)	(−2.256)	(−2.416)
*Politic*	−0.140^***^	−0.120^**^	−0.118^**^
	(−2.882)	(−2.564)	(−2.481)
*Health*	0.708^***^	0.742^***^	0.733^***^
	(28.500)	(29.993)	(29.588)
*Town*	−0.065^*^	−0.041	−0.064^*^
	(−1.708)	(−1.082)	(−1.685)
*Education*	0.147^***^	0.150^***^	0.147^***^
	(8.849)	(8.992)	(8.786)
*Marriage*	−0.407^***^	−0.365^***^	−0.363^***^
	(−7.511)	(−6.742)	(−6.773)
*Family*	−0.160^**^	−0.116	−0.170^**^
	(−2.052)	(−1.483)	(−2.171)
*Gender*	0.012	0.016	0.021
	(0.316)	(0.411)	(0.538)
*GDP*	−0.230^***^	−0.180^***^	−0.147^**^
	(−3.354)	(−2.664)	(−2.158)
*Entertainment*	−0.200^***^	−0.241^***^	−0.241^***^
	(−2.850)	(−3.547)	(−3.496)
*Medical_ins*	0.480^***^	0.413^***^	0.420^***^
	(8.589)	(7.541)	(7.627)
Observations	8,655	8,655	8,655
*R2*	0.044	0.042	0.041

Robust t-statistics in parentheses.

^***^p < 0.01, ^**^p < 0.05, ^*^p < 0.1.

Furthermore, this study posits that the growth of the digital economy could boost older adults' happiness by expanding their social capital. According to the findings from Table 7 Column (2), there is a significant positive impact (coefficient 0.122, *p* < 0.1) derived from the interaction of the digital economy level and social capital, supporting Hypothesis 3. Thus, the digital economy contributes to larger social capital, enhancing happiness among older adults. Based on social network theory and social support theory, in a context where personal connections dominate, various social relationships widely exist in reality. Individuals, organizations, and other actors formed specific connections with the outside world, creating relatively stable network systems through resource sharing and information exchange (Kwahk and Park, 2016). The digital economy provides older adults with platforms and tools for online social interaction and maintaining relationships. The internet's unique ability for rapid communication enabled remote interactions and the building of professional networks, directly expanding social networks and facilitating social capital transfer (Kraut et al., 1998). Moreover, the digital economy effectively breaks the geographic limitations of social networks, reduces communication costs, enhances convenience in communication and information exchange, and strengthens interpersonal connections, positively impacting the happiness of older adults.

Lastly, this study argues that the digital economy improves the happiness of older adults through enhanced health services. Results from Table 7 Column (3) reveal a positive impact (coefficient 0.547, *p* < 0.01) from the interaction between the digital economy level and health services for older adults, which supports Hypothesis 4. It demonstrates that the digital economy positively affects health service provision, thereby raising the happiness of older adults. The digital economy has accelerated the growth of digital healthcare, such as online medical services and internet hospitals, providing older adults with more convenient and efficient healthcare. Through advanced technologies like 5G, AI, and 3D, older adults can easily monitor their health with real-time data transmitted to doctors. This allows doctors to understand their health conditions promptly and accurately, enabling optimal treatment plans. Digital healthcare not only reduces medical visit times but also improves the accuracy and efficiency of treatments, enhancing the happiness of older adults. Additionally, digital healthcare offers continuous and routine health assessments. Unlike traditional healthcare, which provided only one-time diagnoses, digital healthcare enabled continuous data collection and analysis, delivering more scientific health management solutions that meet older adults' healthcare needs (Xie et al., 2021). Furthermore, the rise of telemedicine, driven by the digital economy, benefits older adults with mobility issues or those living in remote areas. Telemedicine allows them to access professional medical consultations and treatments from home, avoiding long commutes and waiting times. The application of telemedicine significantly contributes to improving their happiness.

## 5 Discussion

This study examines whether local digital economy development is associated with the happiness of older adults in China, using CLASS 2020 matched with a city level digital economy. Across specifications, the estimates point to a stable positive relationship between digital economy development and older adults' happiness. The association also remains when we apply identification and robustness strategies intended to mitigate endogeneity concerns. These results show that digital transformation is not only an economic phenomenon. It can also shape later life wellbeing through everyday experiences, such as access to information, service convenience, and opportunities for social participation in a digital environment.

The central finding aligns with a growing body of research linking digitalization and information technology access to subjective wellbeing. Studies based on multi region data reported that digital access and technology diffusion were associated with higher life satisfaction and wellbeing (Graham and Nikolova, 2013; Kavetsos and Koutroumpis, 2011; Ionescu-Feleagă et al., 2022). Our evidence is consistent with these patterns, while focusing on a group that faces distinct constraints and needs. For older adults, the digital economy can reduce transaction and information costs, ease participation in daily activities, and expand service options. It is especially relevant in settings where online channels increasingly complement or substitute offline services.

The study adds to the literature in ways that address common gaps in prior work. Many studies proxy digital development with a single indicator, such as internet penetration, or focus primarily on individual internet use. We measure the digital economy at the city level with a composite dimension that integrates internet development and digital financial inclusion. It could reflect a broader digital ecosystem that includes infrastructure and service capability. We also connect this macro digital environment to micro level wellbeing outcomes in an aging population. This combination helps bridge research on digital economy development and research on happiness determinants.

A further contribution is the explicit use of a multidimensional digital divide perspective to interpret heterogeneous wellbeing returns. Digital divide research emphasizes that inequality is not limited to connectivity. It also involves differences in skills, usage patterns, and outcomes [Organisation for Economic Co-operation Development (OECD), 2001; Dewan and Riggins, 2005; Attewell, 2001; Van Dijk and Hacker, 2003; Scheerder et al., 2017]. Our result follows this framework. Older adults who remain offline do not show clear happiness gains from local digital economy development, while older adults who are connected experience markedly larger gains. This pattern is also consistent with evidence that the wellbeing benefits of digital development depend on participation in the digital sphere (Pénard et al., 2013; Lu et al., 2024). Among internet users, higher device proficiency and more frequent use are linked to stronger happiness gains. The purpose of internet use also matters. Uses related to social interaction, entertainment, information acquisition, and investment or consumption are associated with larger gains. It proves that meaningfulness is an important boundary condition for translating digital development into wellbeing improvements among older adults.

The mechanism results support multiple channels linking digital economy development to older adults' happiness. One pathway runs through public services and governance. Digital government reforms reduce administrative burden, improve responsiveness, and strengthen perceptions of safety and service quality, contributing to subjective wellbeing (Andersen, 2009; Eom and Lee, 2022; Madsen et al., 2022). Another pathway operates through social capital. Digital tools lower the cost of maintaining ties and support social interaction across distance; they help older adults sustain networks and social support (Kraut et al., 1998; Kwahk and Park, 2016). A third pathway relates to health services. Digital healthcare and telemedicine reduce access barriers and improve perceived health security, which is closely related to wellbeing, especially in later life (Kotakorpi and Laamanen, 2010; Tavares, 2020; Feng et al., 2024). Hence, the digital economy affects wellbeing not only through market convenience, but also through institutions and social integration.

Overall, the findings indicate that local digital economy development is linked to higher happiness among older adults, but the benefits depend on digital inclusion. Access, skills and usage shape the magnitude of returns. The evidence also supports a multi-channel account that spans governance, social capital, and health services. This integrated perspective helps clarify when and how digital transformation can enhance wellbeing in aging societies.

## 6 Conclusion & implications

Using CLASS 2020 matched with a city level digital economy index, this study finds that older adults living in more digitally developed cities report higher happiness. The association remains robust across alternative specifications and robustness checks, including strategies intended to address endogeneity. At the same time, the benefits are not evenly shared. Older adults who remain offline show no clear happiness gains, while connected older adults experience substantially larger gains. Among internet users, greater device proficiency, more frequent use, and certain purposes of use are linked to stronger happiness gains. The mechanism evidence is consistent with the idea that digital economy development can improve happiness through better digital governance and public service experiences, stronger social capital, and improved access to health services.

These findings have implications for theory part. They position the digital economy as a city level structural correlate of later life wellbeing, complementing the standard focus on individual socioeconomic status and health. They also strengthen the multi-level digital divide perspective by showing that wellbeing returns depend on access, usage, and skills or purpose, not on digital development alone (Attewell, 2001; Van Dijk and Hacker, 2003; Scheerder et al., 2017). The results further explore that public services, social capital, and health-service access are important channels.

The findings have practical relevance for aging societies undergoing digital transition. Digital infrastructure expansion is necessary, but it does not guarantee that older adults will benefit. Inclusion has to be built into policy design. A first priority is inclusive digital access for disadvantaged older adults, alongside broader urban–rural infrastructure upgrades. Subsidies, preferential tariffs, and suitable devices can reduce cost barriers. Community-based onboarding and set-up support reduce practical usage barriers. These measures help ensure that older adults access digital services in daily life.

Access alone is not enough, since capability constraints determine who translate connectivity into wellbeing gains. Policy should therefore shift from facility building to digital skills and usage support. Training should be scenario-based and organized around certain needs, such as using public services, booking medical appointments, and maintaining social communication. Delivery rely on coordinated efforts among local governments, communities, and service providers. Essential applications should also be required to adopt age-friendly design standards, while offline channels remain available for critical services. This combination improves usability and avoids excluding those who cannot fully adopt digital tools during the transition.

An additional priority is deeper integration of digital technologies into key livelihood domains. Specifically, digital public services can be consolidated into integrated, senior-friendly platforms that streamline procedures and reduce repeated paperwork. Communities are expected to support age-friendly social and interest platforms built on community currency information systems. Timebanking can be embedded in these platforms to encourage participation, mutual help, and volunteering among older adults. Such tools reduce social isolation, widen social networks, and enhance social capital. Digital health services should be scaled up as well, including telemedicine and chronic disease management linked to wearable devices and family-doctor support. Digital health literacy programs are also needed so that older adults have the ability to use these services safely and with confidence.

Several limitations qualify these conclusions and point to future research directions. The analysis relies on cross sectional data, which constrains causal interpretation despite the use of instrumental variables and robustness checks. Future work would benefit from longitudinal data, policy shocks to strengthen causal claims. The mechanism variables are based on available survey measures and capture perceptions and reported experiences. Richer measures of actual service use, platform engagement, and objective healthcare utilization would help refine the pathway tests. The study focuses on China, where the scale and speed of digital transformation are distinctive. Comparative evidence across institutional settings would help assess external validity. Future research can also incorporate more direct measures of individual digital literacy and competence to separate the role of city level digital ecosystems from personal digital capacity.

## Data Availability

The original contributions presented in the study are included in the article/supplementary material, further inquiries can be directed to the corresponding authors.

## Footnotes

1 https://www.stats.gov.cn/xxgk/jd/sjjd2020/202601/t20260119_1962338.html.

2 The weights for each component are as follows: 0.153 (internet penetration rate), 0.147 (internet-related output), 0.102 (internet-related employment), 0.115 (mobile phone penetration rate), and 0.481 (digital financial inclusion).
